# Supplementary material for: Resolving the full spectrum of human genome variation using Linked-Reads
Source: Genome Res. 2019 Apr;29(4):635–45. doi: 10.1101/gr.234443.118 (PMC6442396; doi:10.1101/gr.234443.118)
Supplement: Supplemental Material [file supp_gr.234443.118_Supplemental_longranger-2.2.zip › longranger-2.2.tar.bz2!/lr2.2-public/lib/stan/cmdstan/stan/doc/api/html/promoter_8hpp_source.html]

Stan: src/stan/math/matrix/promoter.hpp Source File


|  |  |
| --- | --- |
|  | Stan  2.5.0  probability, sampling & optimization |


- Main Page
- Related Pages
- Namespaces
- Classes
- Files

- File List
- File Members

All Classes Namespaces Files Functions Variables Typedefs Enumerations Enumerator Friends Macros Pages

- src
- stan
- math
- matrix

promoter.hpp

Go to the documentation of this file.

1 #ifndef STAN\_\_MATH\_\_MATRIX\_\_PROMOTER\_HPP

2 #define STAN\_\_MATH\_\_MATRIX\_\_PROMOTER\_HPP

3

4 #include <vector>

5 #include <stan/math/matrix/Eigen.hpp>

6

7 namespace stan {

8

9  namespace math {

10  // from input type F to output type T

11

12  // scalar, F != T (base template)

13  template <typename F, typename T>

14  struct promoter {

15  inline static void promote(const F& u, T& t) {

16  t = u;

17  }

18  inline static T promote\_to(const F& u) {

19  return u;

20  }

21  };

22  // scalar, F == T

23  template <typename T>

24  struct promoter<T,T> {

25  inline static void promote(const T& u, T& t) {

26  t = u;

27  }

28  inline static T promote\_to(const T& u) {

29  return u;

30  }

31  };

32

33  // std::vector, F != T

34  template <typename F, typename T>

35  struct promoter<std::vector<F>, std::vector<T> > {

36  inline static void promote(const std::vector<F>& u,

37  std::vector<T>& t) {

38  t.resize(u.size());

39  for (size\_t i = 0; i < u.size(); ++i)

40  promoter<F,T>::promote(u[i],t[i]);

41  }

42  inline static std::vector<T>

43  promote\_to(const std::vector<F>& u) {

44  std::vector<T> t;

45  promoter<std::vector<F>,std::vector<T> >::promote(u,t);

46  return t;

47  }

48  };

49  // std::vector, F == T

50  template <typename T>

51  struct promoter<std::vector<T>, std::vector<T> > {

52  inline static void promote(const std::vector<T>& u,

53  std::vector<T>& t) {

54  t = u;

55  }

56  inline static std::vector<T> promote\_to(const std::vector<T>& u) {

57  return u;

58  }

59  };

60

61  // Eigen::Matrix, F != T

62  template <typename F, typename T, int R, int C>

63  struct promoter<Eigen::Matrix<F,R,C>, Eigen::Matrix<T,R,C> > {

64  inline static void promote(const Eigen::Matrix<F,R,C>& u,

65  Eigen::Matrix<T,R,C>& t) {

66  t.resize(u.rows(), u.cols());

67  for (int i = 0; i < u.size(); ++i)

68  promoter<F,T>::promote(u(i),t(i));

69  }

70  inline static Eigen::Matrix<T,R,C>

71  promote\_to(const Eigen::Matrix<F,R,C>& u) {

72  Eigen::Matrix<T,R,C> t;

73  promoter<Eigen::Matrix<F,R,C>,Eigen::Matrix<T,R,C> >::promote(u,t);

74  return t;

75  }

76  };

77  // Eigen::Matrix, F == T

78  template <typename T, int R, int C>

79  struct promoter<Eigen::Matrix<T,R,C>, Eigen::Matrix<T,R,C> > {

80  inline static void promote(const Eigen::Matrix<T,R,C>& u,

81  Eigen::Matrix<T,R,C>& t) {

82  t = u;

83  }

84  inline static Eigen::Matrix<T,R,C> promote\_to(const Eigen::Matrix<T,R,C>& u) {

85  return u;

86  }

87  };

88

89  }

90 }

91

92

93 #endif

stan::math::promoter< T, T >::promote

static void promote(const T &u, T &t)

**Definition:** promoter.hpp:25

stan::math::promoter< std::vector< T >, std::vector< T > >::promote

static void promote(const std::vector< T > &u, std::vector< T > &t)

**Definition:** promoter.hpp:52

stan::math::promoter< Eigen::Matrix< F, R, C >, Eigen::Matrix< T, R, C > >::promote

static void promote(const Eigen::Matrix< F, R, C > &u, Eigen::Matrix< T, R, C > &t)

**Definition:** promoter.hpp:64

stan::math::promoter< std::vector< F >, std::vector< T > >::promote

static void promote(const std::vector< F > &u, std::vector< T > &t)

**Definition:** promoter.hpp:36

stan::math::promoter< T, T >::promote\_to

static T promote\_to(const T &u)

**Definition:** promoter.hpp:28

stan::math::promoter< Eigen::Matrix< T, R, C >, Eigen::Matrix< T, R, C > >::promote

static void promote(const Eigen::Matrix< T, R, C > &u, Eigen::Matrix< T, R, C > &t)

**Definition:** promoter.hpp:80

Eigen.hpp

stan::math::promoter::promote

static void promote(const F &u, T &t)

**Definition:** promoter.hpp:15

stan::math::promoter::promote\_to

static T promote\_to(const F &u)

**Definition:** promoter.hpp:18

stan::math::promoter< std::vector< F >, std::vector< T > >::promote\_to

static std::vector< T > promote\_to(const std::vector< F > &u)

**Definition:** promoter.hpp:43

stan::math::promoter

**Definition:** promoter.hpp:14

stan::math::promoter< std::vector< T >, std::vector< T > >::promote\_to

static std::vector< T > promote\_to(const std::vector< T > &u)

**Definition:** promoter.hpp:56

stan::math::promoter< Eigen::Matrix< F, R, C >, Eigen::Matrix< T, R, C > >::promote\_to

static Eigen::Matrix< T, R, C > promote\_to(const Eigen::Matrix< F, R, C > &u)

**Definition:** promoter.hpp:71

stan::math::promoter< Eigen::Matrix< T, R, C >, Eigen::Matrix< T, R, C > >::promote\_to

static Eigen::Matrix< T, R, C > promote\_to(const Eigen::Matrix< T, R, C > &u)

**Definition:** promoter.hpp:84


---

[ Stan Home Page ]

*© 2011–2014,
Stan Development Team.*
